# Terahertz Laser Pulse Boosts Interlayer Spin Transfer in Two-Dimensional van der Waals Magnetic Heterostructures

**DOI:** 10.1021/acs.jpclett.3c03000

**Published:** 2023-12-07

**Authors:** Min Li, Junjie He

**Affiliations:** † Department of Physical and Macromolecular Chemistry, Faculty of Science, Charles University, Prague 12843, Czech Republic

## Abstract

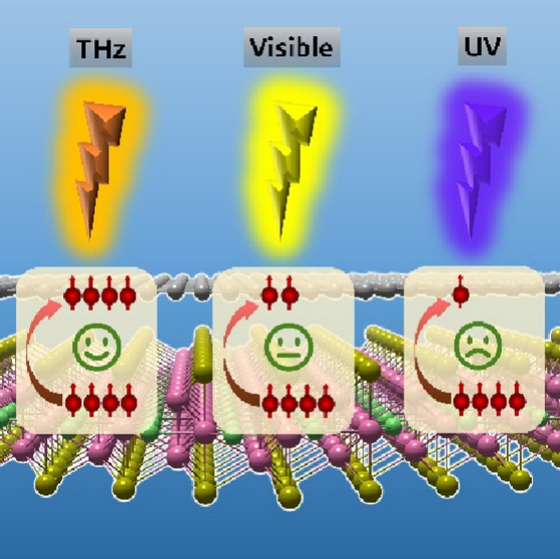

Light-induced ultrafast dynamics in two-dimensional (2D) magnetic systems demonstrate substantial advancements in spintronics. Here, using the real-time time-dependent density functional theory (rt-TDDFT), we applied laser pulses with various frequencies, from terahertz (THz) to optical pulse, to systematically study the interlayer spin transfer dynamics in 2D van der Waals nonmagnetic–ferromagnetic heterostructures, including graphene-Fe_3_GeTe_2_ (Gr/FGT) and silicene-Fe_3_GeTe_2_ (Si/FGT). Our results demonstrate that low-frequency THz pulses are particularly effective in facilitating interlayer spin injection from the ferromagnetic FGT layers to the Si or Gr layers. On the contrary, high-frequency optical pulses exhibit a minimal influence on this process. Such an effect is attributed to the low-frequency THz pulses inducing in-phase oscillations of the electron charge density around atomic centers, leading to a highly efficient interlayer spin transfer. Our results provide a new insight into ultrafast THz radiation control intralayer spin transfer and magnetic proximity dynamics in the 2D limit.

Light-induced ultrafast spin dynamics of magnetic materials is an essential basis for spintronics and magnetic storage technology because they enable rapid manipulation and switching of magnetic states on femtosecond, even attosecond time scales.^1,2^ Previous studies mainly focused on the demagnetization induced by optical laser pulses, leading to the proposal of various mechanisms on ultrafast demagnetization dynamics, including spin–orbit coupling effect, spin-flip scattering, spin polarization transport, etc.^3−5^ Nonetheless, the high energy associated with optical laser pulses presents significant challenges such as overheating, potential damage, or structural alterations to the sample, all of which could dramatically affect the efficiency and repeatability of demagnetization processes. To overcome these issues, a new excitation method, terahertz (THz) radiation, has emerged in recent years.^6^ THz radiation, with its low energy characteristics, offers the potential for resonant excitation through spin precession or charge oscillation, facilitating precise control over the magnetization state.^7−9^ However, it is noted that the theoretical understanding of the THz radiation-induced demagnetization process is still in a preliminary stage.

Two-dimensional (2D) magnetic materials were first discovered in exfoliated van der Waals (vdW) CrI_3_ and CrGeTe_3_ crystals, attracting great interest for controlling spin in the 2D limit.^10,11^ Following this, 2D Ising ferromagnets, Fe_3_GeTe_2_ (FGT), were reported in experiments,^12,13^ which shows a high Curie temperature (about 230 K), pronounced perpendicular magnetic anisotropy, and gate tunable magnetism.^14^ Further developments revealed that ultrafast light could serve as an efficacious tool for manipulating the spin dynamics in 2D magnetic materials. Liu et al.,^15^ for instance, successfully employed laser pulses to manipulate the magnetization, magnetic anisotropy, and Curie temperature of ferromagnetic FGT film. Similarly, Zhang et al.^16^ demonstrated all-optical magnetization switching in the atomically thin ferromagnetic semiconductor of CrI_3_ by using circularly polarized light pulses. Despite these experimental advances, theoretical understanding for the laser-pulse-induced ultrafast process in 2D magnetic materials is a relatively unexplored field.

2D magnets have been flexibly integrated into van der Waals heterostructures (vdWH), showcasing remarkable potential for applications in spintronics devices. A magnetic proximity effect (MPE) will occur when 2D ferromagnets come into contact with nonmagnetic (NM) materials, such as graphene, transition-metal dichalcogenides (TMDs), and topological insulators (e.g., Bi_2_Se_3_). This MPE will give rise to a variety of exotic quantum properties and pave the way for innovative device applications, including valley polarization and magnetic tunnelling junctions.^17−20^ A prime example is the graphene/FGT vdWH, which can achieve current-assisted magnetization inversion in spintronics devices.^21^ In addition, the interface interaction between FGT and 2D nonmagnetic materials also significantly influences their electronic structure and interlayer spin transfer,^22,23^ providing a platform for further revealing novel physical phenomena in 2D heterojunctions. Seyler et al.^24^ have demonstrated that a laser pulse can manipulate the valley polarization by adjusting the MPE and interlayer spin transfer. However, the dynamics of interlayer spin transfer remains incompletely understood, necessitating further theoretical investigation.

The optically induced intersite spin transfer (OISTR) effect enables light to redistribute spin in multicomponent magnetic compounds, facilitating us toward the optical control of spin on the attosecond time scale.^25^ Several experimental works have confirmed that the OISTR effect occurs across diverse systems, encompassing metallic multilayers and Heusler compounds.^26−30^ While there has been substantial investigation into photoinduced spin dynamics, along with the associated mechanisms within 2D magnets and vdWH,^31,32^ a lacuna remains in our understanding of how laser pulse frequency influences spin dynamics in 2D magnetic systems. This is particularly the case in the terahertz range, where the effects have yet to be fully explored and comprehended.

In this Letter, we employ the rt-TDDFT simulations to explore the impact of diverse laser pulse frequencies, encompassing terahertz and optical ranges, on the interlayer spin and charge transfer dynamics within Gr/FGT and Si/FGT vdWH. We elucidate that low-frequency terahertz pulses are markedly more efficacious in facilitating spin injection and demagnetization across both Gr/FGT and Si/FGT heterostructures, compared to their high-frequency optical pulse counterparts. We performed a comprehensive analysis to unravel the underlying physical mechanisms.

## Ground States Structural, Magnetic, and Electronic Properties

**Figure 1:**
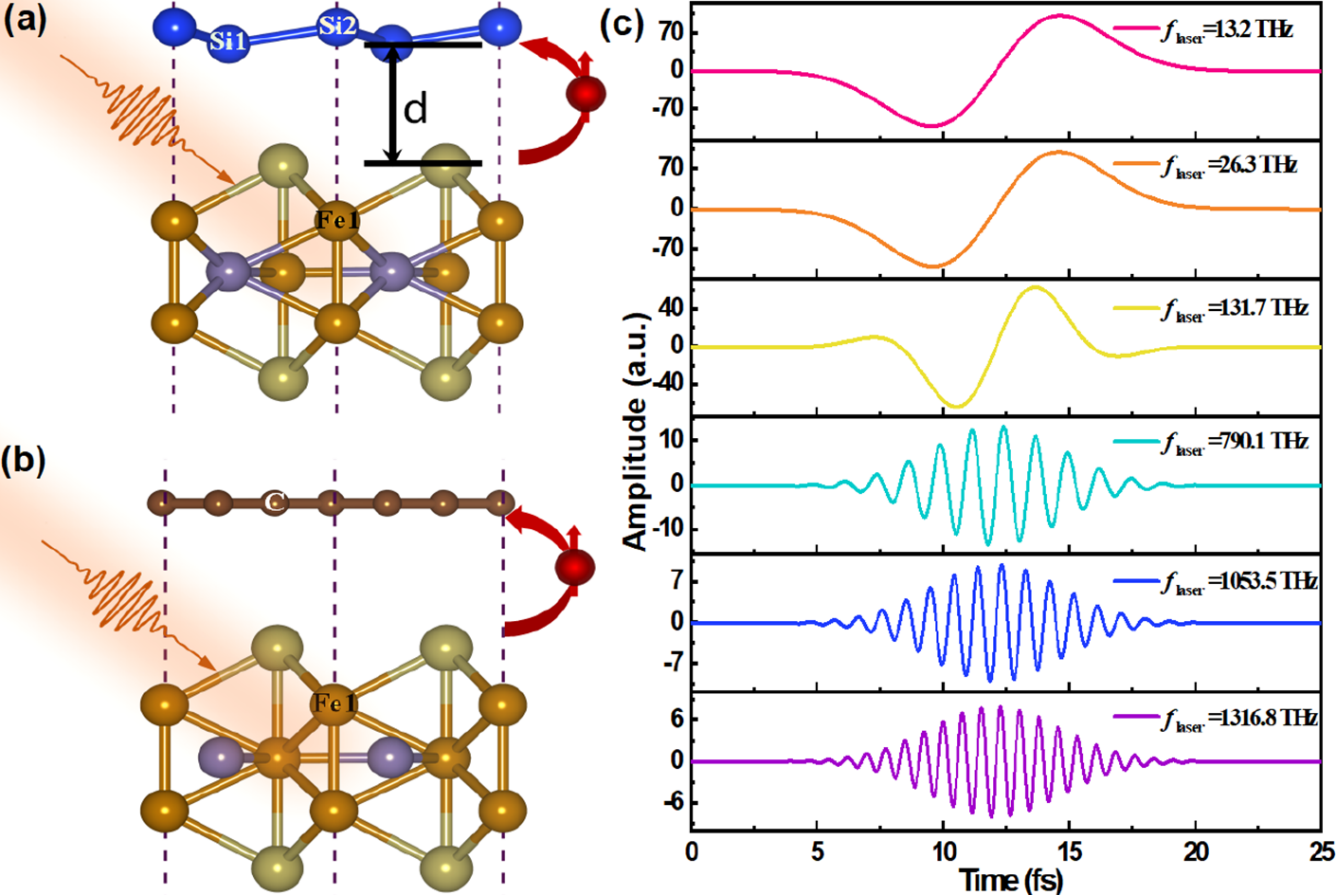
Depiction of the van der Waals heterostructure model and incident pulses. (a) Illustrated geometric configurations of Si/FGT and Gr/FGT heterostructures model. Balls with the color of blue, dark brown, brown, tan and dark brow represent Si, C, Fe, Te, and Ge atoms, respectively. (b) Temporal profiles of laser pulses with various frequencies, with each laser frequency distinctly marked.

The geometrical structures of Si/FGT and Gr/FGT vdWH are depicted in Figure 1a. The lattice constant of FGT was optimized to 4.051 Å utilizing the PBE + D2 level, in agreement with previous theoretical investigations.^31^ Subsequently, FGT was interfaced with a 2 × 2 graphene lattice and a 1 × 1 silicene lattice, resulting in lattice constant mismatches of 5.55% and −4.44%, respectively. The equilibrium distances from the FGT surface to Si/FGT and Gr/FGT were calculated to be 2.922 Å for monolayer silicene and 3.356 Å for graphene, respectively (refer to Table S1 for detailed information).

Initially, we conducted DFT calculations to study the ground-state magnetism within Si/FGT and Gr/FGT vdWH. Owing to the MPE of the FGT substrate, notable local magnetic moments were induced, registering at −0.011/0.034 μ_B_ for Si atoms and ranging between 0.0016 and 0.0017 μ_B_ for C atoms. These induced moments are comparable to those observed in the EuO substrate.^33^ Notably, graphene demonstrates a markedly lower proximity-induced magnetic moment in comparison to silicene. The band structures and projected density of states (PDOS) for the Si/FGT and Gr/FGT vdWH are displayed in Figure S1. In this depiction, we can see that the MPE of FGT induced a pronounced alteration of the electronic structure for graphene and silicene. Silicene, in particular, exhibits significant spin splitting and heightened spin polarization, both of which are attributable to the MPE of the FGT substrate. Additionally, the Dirac point in silicene is noticeably destroyed. In contrast, graphene retains its characteristic Dirac point with a slight spin splitting.

## Demagnetization and Interlayer Spin Transfer Dynamics

We next explore the laser pulse frequency-dependent spin dynamics in Si/FGT and Gr/FGT by employing a noncollinear spin version of rt-TDDFT. All rt-TDDFT simulations were performed by all-electron full potential ELK code^34^ (see computational methods in Supporting Information). For these calculations, we utilized a linearly polarized (in-plane polarization) laser pulse with a constant full width at half-maximum (fwhm). The rt-TDDFT simulations were performed across a range of laser pulse frequency (*f*_laser_), specifically at 13.2 THz, 26.3 THz, 131.7 THz, 790.1 THz, 1053.5 THz, and 1316.8 THz, which was plotted in Figure 1b with a fixed power density of 4.78 × 10^12^ W/cm^2^. (Refer to Table S2 for additional information on laser parameters.)

**Figure 2:**
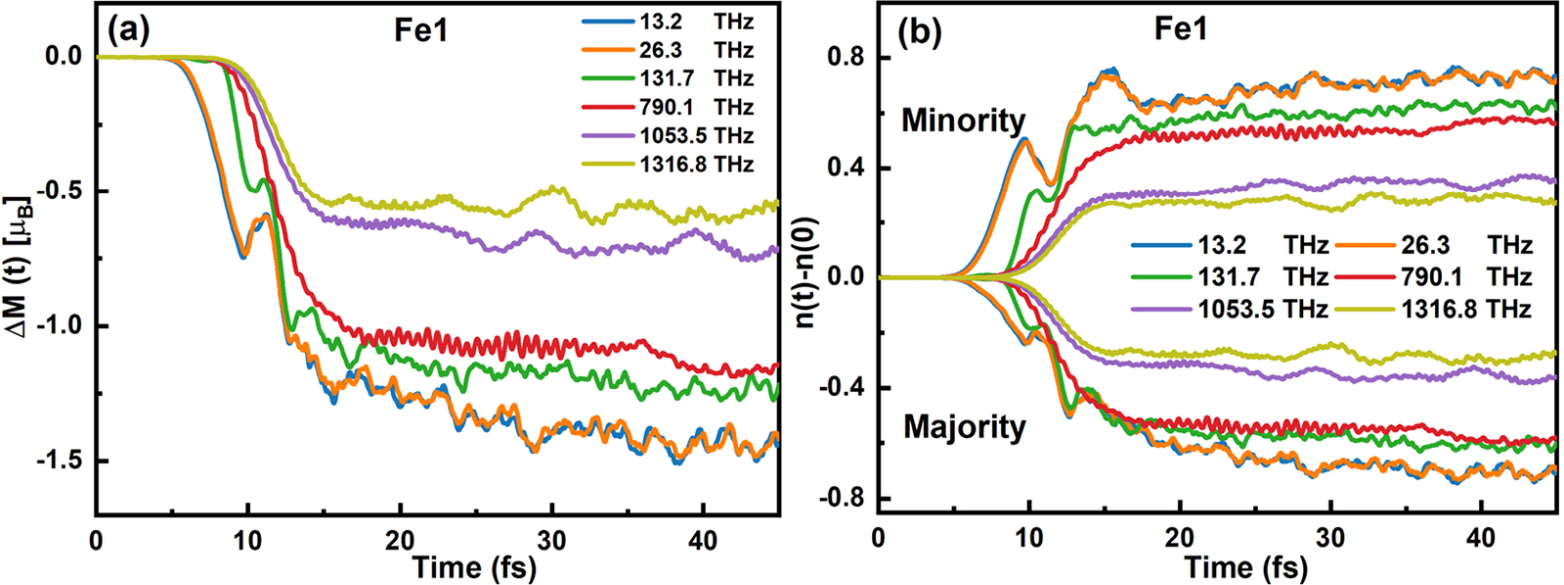
Time-dependent spin dynamics and occupation dynamics. (a) Time dependent dynamics of the local magnetism for the Fe1 of the Si/FGT in different pulses (13.2, 26.3, 131.7, 790.1, 1053.5, and 1316.8 THz). (b) The time dependent change of majority and minority occupations as a function of time (in fs) of Fe1 atoms of the Si/FGT, which is defined as Δ*n* = *n*(*t*) – *n*(0).

We initiated our investigation by examining the real-time evolution of local magnetic moments of Fe1 atoms in Si/FGT under various *f*_laser_ values, as depicted in Figure 2a and Figure S4. For clarity, we refer to the Fe atoms as Fe1 in the subsequent discussions. Our observations revealed that the change in local magnetic moment of Fe1, quantified by Δ*M* = *M*(*t*) – *M*(0), underwent significant and generally strong demagnetization across the different *f*_laser_ conditions. Remarkably, as the *f*_laser_ decreased from 1316.8 THz in the optical range to 13.2 THz in the terahertz range, we noted a pronounced increase in the demagnetization of Fe1, ranging from 0.4 μ_B_ to 0.8 μ_B_. Specifically, at a higher *f*_laser_ of 1316.8 THz, the demagnetization process of Fe1 was observed to initiate rapidly, lasting for about 5 fs, before saturating at approximately −0.5 μ_B_. Conversely, at a lower *f*_laser_ of 13.2 THz, the demagnetization exhibited a two-step process: (i) a rapid demagnetization occurring between 5 and 12 fs, where the magnetic moment decreased from 0 to approximately −1.1 μ_B_, and (ii) a subsequent slower demagnetization phase extending from 12 to 37 fs, eventually saturating at around −1.4 μ_B_. This behavior is primarily attributed to spin-flip processes induced by spin–orbit coupling (SOC).^35,36^ We demonstrated the demagnetization dynamics of Fe1, specifically examining its *x*, *y*, and *z* components at both low and high frequencies, as illustrated in Figure S2. Our findings reveal that, irrespective of the frequency (whether low or high), the demagnetization of Fe1 predominantly occurs in the *z* direction with minimal impact on the *x* and *y* components. Consequently, we omitted the consideration of the *x* and *y* components in our analysis of the spin dynamics.

We note that there is no clear linear relation between the demagnetization rate and frequency. Instead, the demagnetization rate will strongly depend on the fluence of the laser pulse. Additionally, our analysis revealed that lower frequencies show a stronger effect on spin-flip processes. Interestingly, analogous phenomena were observed in the Gr/FGT heterojunction, as illustrated in Figure S3. This indicates that the demagnetization is not closely related to the substrate type but is predominantly influenced by the frequency of the applied laser pulse.

To gain a deeper understanding of demagnetization dynamics under various pulse frequencies, we investigated the time-dependent changes (denoted as Δ*n*(*t*)) in the occupation of Fe1 atoms. This parameter is defined as the differences between the time-resolved occupation function, *n*(*t*), at any given time *t*, and the initial occupation in unexcited Si/FGT or Gr/FGT vdWH, formulated as Δ*n*(*t*) = *n*(*t*) – *n*(0). Figure 2b depicts the evolution of the number of majority and minority occupations of Fe1 in Si/FGT. A reduction in the majority occupations coupled with an increase in the minority occupations corresponds to the demagnetization of the Fe1 moment. It is particularly evident that lower *f*_laser_ pulses induce stronger charge excitation in both majority and minority channels compared to their high *f*_laser_ counterparts. For example, at an *f*_laser_ of 13.2 THz, the majority channel undergoes a significant loss of occupations (Δ*n* = −0.7), eventually reaching a state of saturation, whereas the minority channel experiences a corresponding gain in occupations (Δ*n* = 0.7), also reaching saturation. In contrast, at a higher *f*_laser_ of 1316.8 THz, the majority channel exhibits a more modest loss of occupations (Δ*n* = −0.25) before saturation, while the minority channel sees an occupations gain of Δ*n* = 0.25, subsequently reaching saturation as well. In summary, the demagnetization process is predominantly driven by photoinduced spin-selective charge transfer across both the majority and the minority states of the Fe1 atoms.

**Figure 3:**
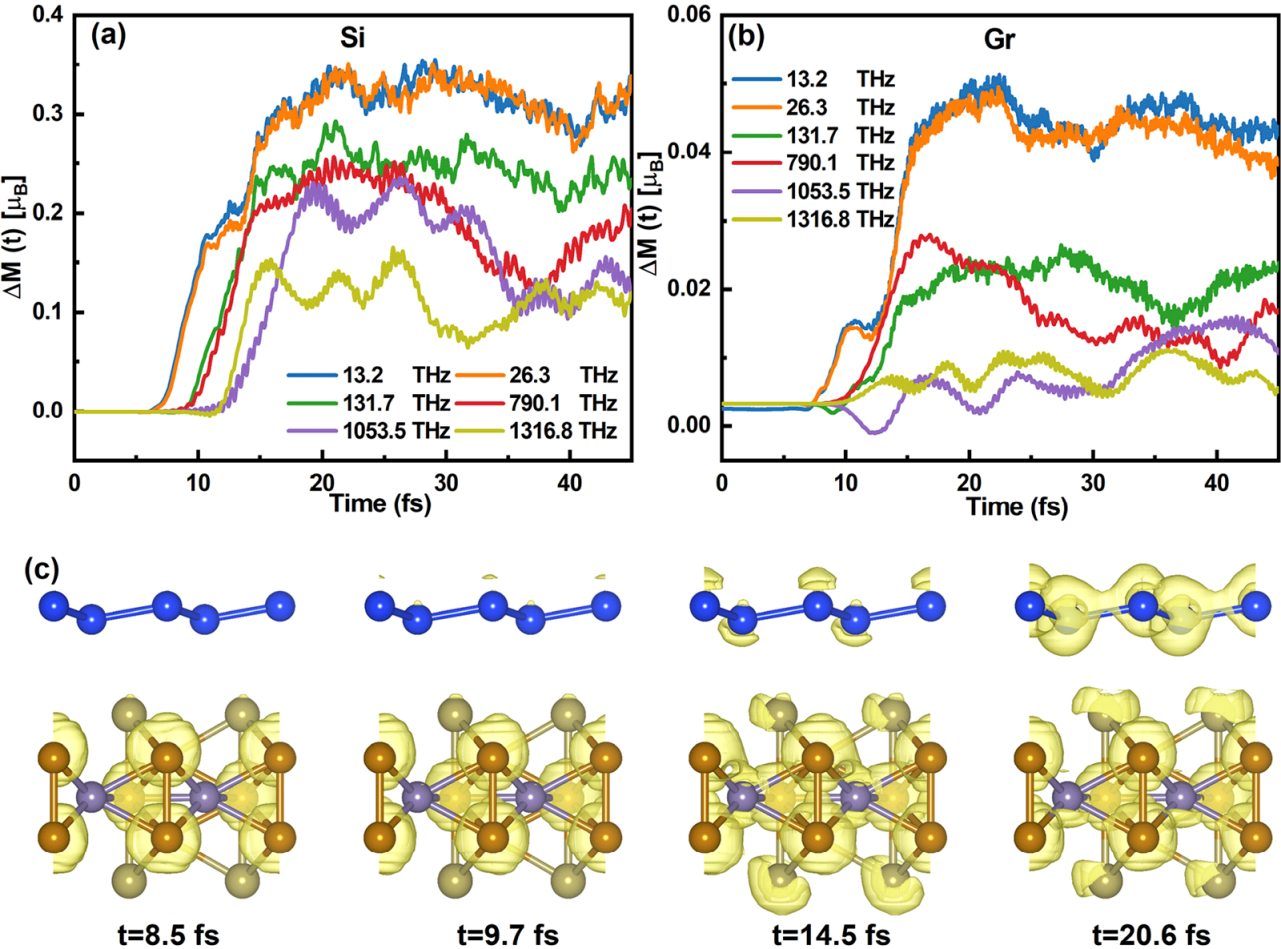
Time-dependent spin dynamics and spin density. (a) Time dependent dynamics of the local magnetic moment for silicene in Si/FGT (a) and graphene (b) in different pulses at 13.2, 26.3, 131.7, 790.1, 1053.5, and 1316.8 THz. (c) Snapshots of the spin density at *t* = 8.5 fs, *t* = 9.7 fs, *t* = 14.5 fs, and *t* = 20.6 fs. The isosurface is set to 0.004 e/Å^3^. Yellow represents spin-up density. The magnetic moment is per unit cell.

Next, we will explore interlayer spin transfer dynamics from the FGT layer to the NM layer. As illustrated in Figure 3a, there is an obvious trend observed in the magnetic moment of silicene: as the *f*_laser_ decreases from 1316.8 THz to 13.2 THz, the magnetic moment of silicene exhibits a gradual increase, ranging from 0.12 μ_B_ to 0.35 μ_B_. As for Gr/FGT, the magnitude of spin transfer is obviously smaller than silicene’s. This is derived from the number of unoccupied states of silicene being larger than that in graphene, which can accept the spin injection from the FGT substrate, which is in agreement with our previous results.^31^ But, the low *f*_laser_ still induced significant spin transfer compared to the high *f*_laser_ as shown in Figure 3b

To gain further insights into spin injection processes from FGT to the NM layers, we examined the time evolution of magnetization density within real space. As depicted in Figure 3c, at a *f*_laser_ of 13.2 THz, there is a discernible gradual transfer of the magnetic moment from the Fe atoms to the adjacent interfacial Te atoms, subsequently extending to the silicene layer. Specifically, this spin injection progress occurs between 8.5 and 20 fs, completing the injection from the Fe to the Si layer in around 11 fs. Furthermore, based on the analysis of the time-dependent changes in the occupation of Si1 of Si/FGT in Figure S5, it is observed that the increased magnetic moment of these atoms can be attributed to the enhancement of majority spins and the reduction of minority spins in NM atoms. These observations unequivocally demonstrate that the spin injections and subsequent demagnetization phenomena within NM-FGT heterostructures are the result of spin selective charge transfer in both minority and majority states. Additionally, it is evident that the spin injection and demagnetization processes within NM-FGT heterostructures are highly susceptible to variations in the laser pulse frequency, thus indicating that this laser parameter can also be applied to optimize and control the spin injection process.

## Mechanism of THz Boosting Interlayer Spin Transfer

**Figure 4:**
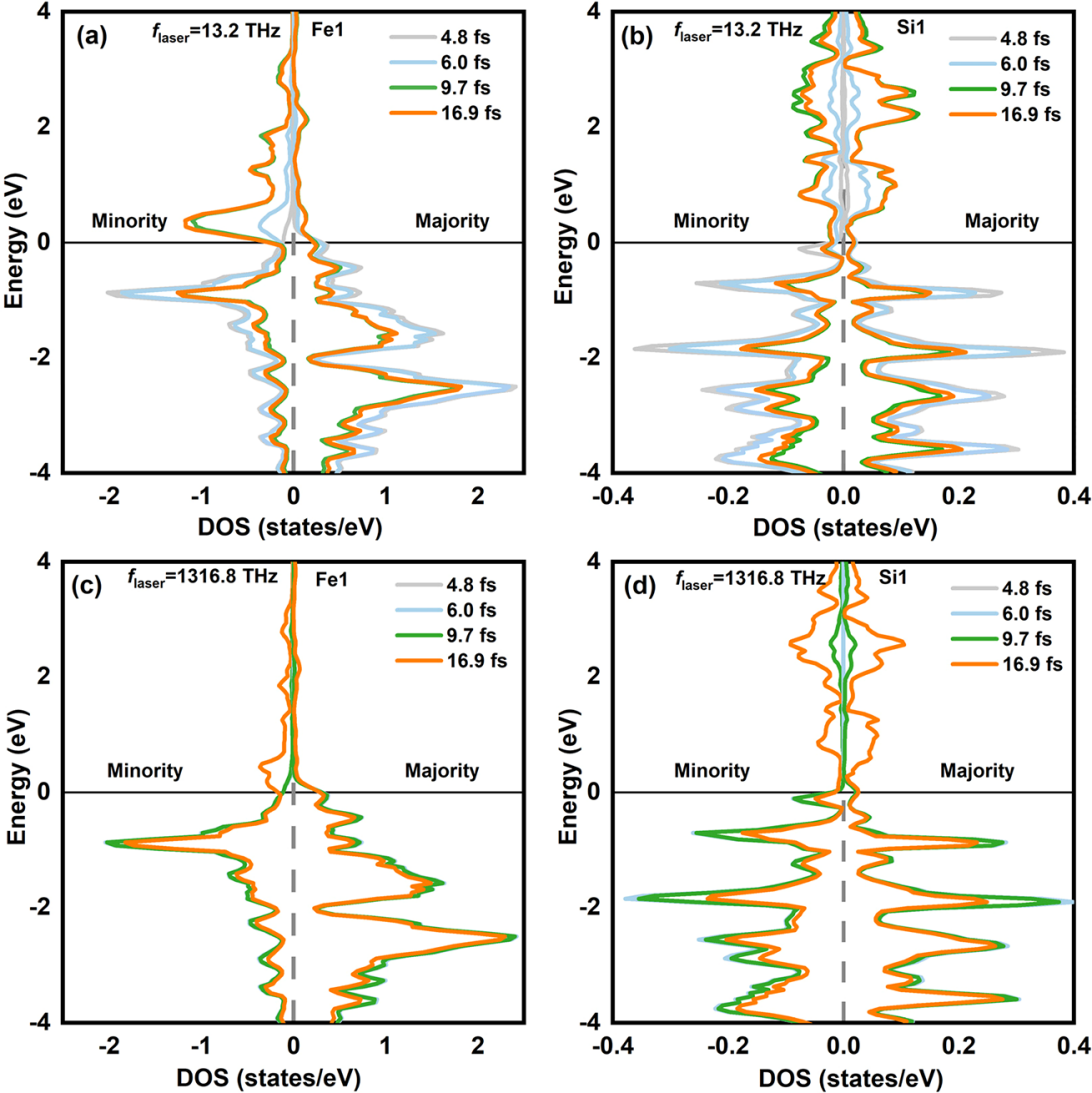
Differences in the time-resolved density of states (DOS). The DOS (in state/eV) for the low *f*_laser_ 13.2 Thz of Fe1 (a) and Si1 (b) in Si/FGT. For the high *f*_laser_ 1316.8 THz of Fe1 (c) and Si1 (d) in Si/FGT. Snap-shots at four different times (4.8, 6, 9.7, and 16.9 fs) are shown. Spin-up and -down correspond to the majority and minority, respectively. The solid black line is the Fermi energy level.

Given that the demagnetization and spin injection processes are predominantly influenced by frequency, our primary analytical focus is on contrasting the time dependent densities of states (DOS) for Fe1 and Si1 in both low- and high-frequency regimes, as showcased in Figure 4. Figure 4a illustrates the time dependent DOS of Fe1 at a *f*_laser_ of 13.2 THz. It is evident that the DOS distribution of Fe1 experiences significant changes over the time span from 4.8 to 16.9 fs. During this interval, there is a gradual decrease in the number of spin-up states in Fe1, while the number of spin-down states steadily increases. This behavior can be attributed to the presence of empty states in Si1 that are capable of accepting electrons in the spin-up direction, whereas there are no such available empty states in the spin-down direction, as illustrated in Figure 4b. Conversely, at a higher *f*_laser_ of 1316.8 THz, as depicted in Figure 4c,d, this change of time-resolved DOS is less pronounced and mainly occurs between 9.7 and 16.9 fs. During this period, there is a slight increase in the number of spin-down electrons. Consequently, the demagnetization effect is not as prominent at high frequencies. As for time dependent DOS for Si1 in Figure 4b and d, we also observe that the difference between the changes in the majority and minority states is remarkably slight. Hence, it can be deduced that lower frequencies induce a more substantial excitation of electrons in both majority and minority spin channels, culminating in a more pronounced spin-selective charge transfer. This transfer encompasses a loss of the majority in Fe1 and an acceptance by the majority in Si1.

**Figure 5:**
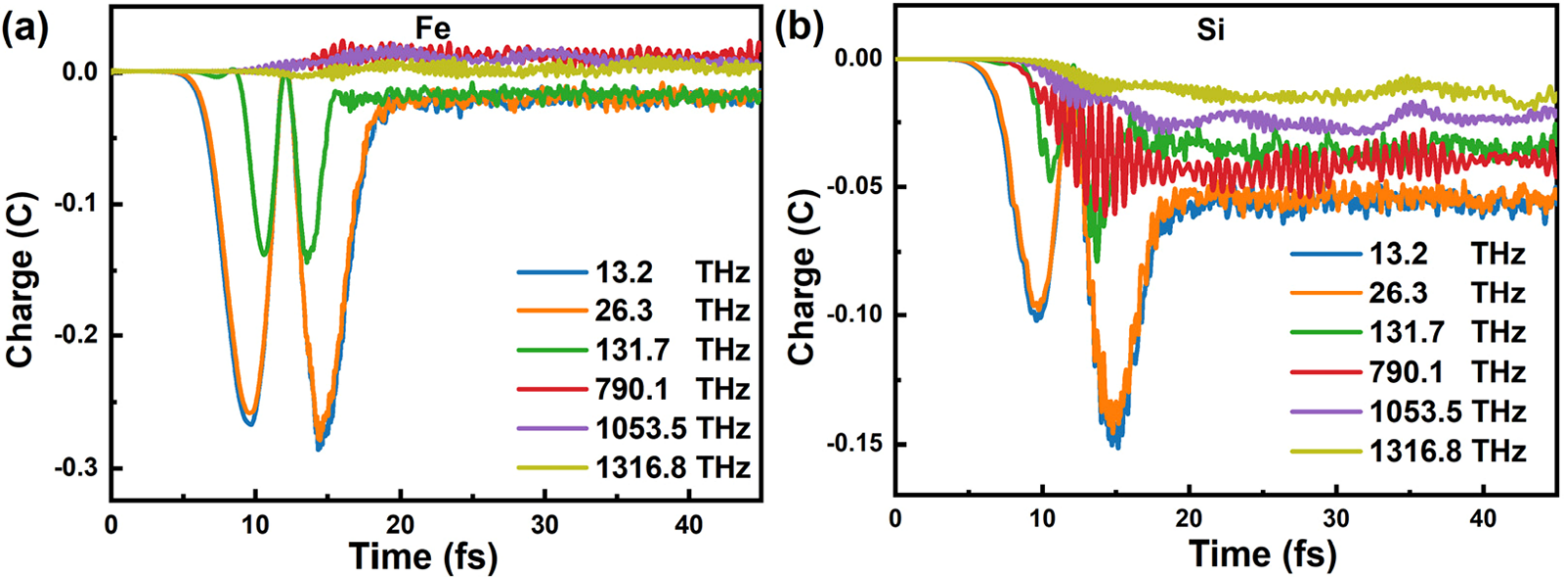
Time-dependent charge dynamics. Charge density for (a) Fe in Si/FGT and (b) Si in Si/FGT. Snapshots at six different frequencies (13.2, 26.3, 131.7, 790.1, 1053.5, and 1316.8 THz) are shown, respectively.

In addition, to further analyze the mechanism of why the Thz radiation significantly boosts the interlayer spin transfer, we focused on the charge effects of unit cell Fe and Si in the Si/FGT at different frequencies, as shown in Figure 5. We observe two distinct peaks of charge oscillations as the frequency decreases in Fe, especially at a low 13.2–26.3 THz frequency. The peak of the charge (Δcharge(*t*) = charge(*t*) – charge(0)) is −0.28. With increasing frequency, this amplitude of oscillation rapidly diminishes and there is almost no change in the high frequency pulse with 1053.5 and 1316.8 THz as shown in Figure 5a. A similar phenomenon is also observed in Si and C in Figure 5b and Figure S5, respectively. These distinct charge oscillations can be attributed to the differing capabilities of optical and THz radiation to drive intra-atomic charge excitations from core electrons. The optical field oscillates on a much faster time scale (∼fs), surpassing the natural speed of electronic motion in solids, resulting in the electrons’ inability to follow the rapid reversal of the light field.^37^ On the contrary, THz field oscillations are much slower, with the direction reversing over 15 fs (refer to Figure 1c), a time scale sufficient for electrons to respond to a distorted electrostatic potential and relax accordingly. Therefore, oscillations of the THz field induce more significant in-phase charge density oscillations than high-frequency radiation, resulting in more effective interlayer spin transfer. A similar phenomenon has also been observed in ferromagnetic metals such as Fe, Co, and Ni.^37^

It is crucial to highlight that our simulations mainly explored the early spin dynamics occurring within the initial 50 fs following the laser pulse. This time scale is a pure optical process without the consideration of spin rotation and the movement of magnetic atoms. However, it is essential to note that in the subsequent time range of 50–100 fs, electron–phonon coupling (EPC) comes into play, significantly impacting the spin dynamics and the magnetic stability within 2D magnetic heterostructures.^38^ Unfortunately, such processes are currently beyond the scope of our TDDFT simulations. However, how EPC influences the interlayer spin transfer dynamics remains an open question for further exploration. In experiments, our work can be validated using ultrafast extreme ultraviolet (EUV) high harmonic pulses and time-resolved magnetic circular dichroism (MCD), both extensively utilized for investigating element-specific spin dynamics in magnetic multilayered films.^26−28^

In summary, we have employed DFT and rt-TDDFT simulations to investigate the ground-state properties and laser-induced spin dynamics within nonmagnetic–ferromagnetic (Si/FGT and Gr/FGT) vdW heterostructures. Our DFT calculations reveal the induction of spin-polarized electronic structures and a significant proximity effect in graphene and silicene owing to the influence of the FGT substrate. Following this, we utilized rt-TDDFT to comprehensively analyze the interlayer spin transfer dynamics and demagnetization phenomena triggered by laser pulses spanning a broad frequency range from THz to the optical regime. Our findings demonstrated that low-frequency THz pulses are particularly effective in facilitating interlayer spin injection from the ferromagnetic FGT layers to the NM Si or Gr layers. However, this process is hardly influenced by high-frequency optical pulses. On the contrary, high-frequency optical pulses exhibit a minimal influence on this process. This observed preference for low-frequency THz pulses is attributed to their induction of in-phase oscillations of the electron charge density around atomic centers, leading to highly efficient interlayer spin transfer. Overall, our research highlights the potential of THz radiation in exploring and manipulating interlayer spin transfer dynamics and magnetic proximity in 2D magnetic vdW heterostructures.

## Supporting Information Available

Computational details, structure parameters and initial magnetic moment, projected band structure and density of state, laser pulse parameters, changes in the magnetic moment of Gr/FGT, time-dependent atomic magnetic moment, time evolution on the charge density of Si/FGT (PDF)
