# Supplementary material for: Terahertz Laser Pulse Boosts Interlayer Spin Transfer in Two-Dimensional van der Waals Magnetic Heterostructures
Source: J Phys Chem Lett. 2023 Dec 7;14(50):11274–80. doi: 10.1021/acs.jpclett.3c03000 (PMC10749471; doi:10.1021/acs.jpclett.3c03000)
Supplement: Supplementary file 1 — jz3c03000_si_001.pdf [file jz3c03000_si_001.pdf]

Supplementary Materials **"Terahertz Laser Pulse Boosts Interlayer Spin Transfer in Two-Dimensional van der Waals Magnetic Heterostructures"**

Min Li [1], Junjie He [1, *]

[1] Department of Physical and Macromolecular Chemistry, Faculty of Science, Charles University, Prague 12843, Czech Republic

Corresponding Author:

Dr. Junjie He

E-mail: junjie.he.phy@gmail.com

Computational Methods

All of the calculations for the ground state were executed using the Vienna Ab initio Simulation Package (VASP) [1]. Within the framework of the generalized gradient approximation, the Perdew-Burke-Ernzerhof (PBE) functional[2] was employed to handle the exchange-correlation interaction. To account for the electron-ion interaction, the projector-augmented wave method was employed [3, 4]. For the purposes of geometry optimization and electronic structure calculations, an energy cutoff of 500 eV and a Monkhorst-Pack 9x9x1 k-mesh grid were utilized [5]. The lattice constants and atomic positions were fully relaxed until the atomic forces were less than 0.01 eV $Å^{-1}$. The convergence criterion for electron relaxation was set at 10^{-6} eV. In order to account for the van der Waals weak interaction between nonmetal and metal materials, the Grimme DFT-D3 approach was employed [6]. A vacuum region of 15 Å was introduced along the out-of-plane direction to prevent interaction between neighboring periodic units.

To investigate the dynamics of spin induced by laser pulses, we conducted calculations using real-time time-dependent density functional theory (rt-TDDFT). The time-evolving state functions were obtained by solving the time-dependent Kohn-Sham (KS) equation, as shown in equation (1).

$$i\frac{\partial\psi_j(\mathbf{r},t)}{\partial t} = \left[\frac{1}{2}\left(-i\nabla + \frac{1}{c}\mathbf{A}_{\mathbf{ext}}(t)\right)^2 + v_s(\mathbf{r},t) + \frac{1}{2c}\sigma\cdot\mathbf{B}_{\mathbf{s}}(\mathbf{r},t) + \frac{1}{4c^2}\sigma\cdot\left(\nabla v_s(\mathbf{r},t)\times -i\nabla\right)\right]\psi_j(\mathbf{r},t) \quad (1)$$

In this equation, the $\mathbf{A}_{\mathbf{ext}}(t)$ and σ represent the vector potential and Pauli matrices, respectively. The KS effective potential $v_s(\mathbf{r},t) = v_{ext}(\mathbf{r},t) + v_H(\mathbf{r},t) + v_{xc}(\mathbf{r},t)$ is composed of three terms: the external potential v_{ext}, the classical Hartree potential v_H, and the exchange-correlation (XC) potential v_{xc}. The KS magnetic field can be expressed as $\mathbf{B}_{\mathbf{s}}(\mathbf{r},t) = \mathbf{B}_{\mathbf{ext}}(\mathbf{r},t) + \mathbf{B}_{\mathbf{xc}}(\mathbf{r},t)$, where $\mathbf{B}_{\mathbf{ext}}$ and $\mathbf{B}_{\mathbf{xc}}$ represent the magnetic field of the applied laser pulse and XC magnetic field, respectively. The last term in equation (1) represents the spin-orbit coupling (SOC) term. Throughout the rt-TDDFT simulations, the motion of the nuclei was kept frozen at all times.

The flow of the spin current governs the spin dynamics of NM/FGT heterostructures, and this flow can be described using the spin current density tensor. The equation that defines the motion of magnetization density $\mathbf{J}(\mathbf{r})$ in relation to the spin current density tensor can be established as follows:

$$i\frac{\partial\psi_j(\mathbf{r},t)}{\partial t} = \left[\frac{1}{2}\left(-i\nabla + \frac{1}{c}\mathbf{A}_{\mathbf{ext}}(t)\right)^2 + v_s(\mathbf{r},t) + \frac{1}{2c}\sigma\cdot\mathbf{B_s}(\mathbf{r},t) + \frac{1}{4c^2}\sigma\cdot\left(\nabla v_s(\mathbf{r},t)\times -i\nabla\right)\right]\psi_j(\mathbf{r},t) \quad (1)$$

To calculate the photoinduced dynamics, we utilized a fully non-collinear spin version of rt-TDDFT and a full-potential augmented plane-wave ELK code[7], resulting in a highly comprehensive analysis. Our calculations were conducted on a regular mesh in a k-space of $8 \times 8 \times 1$, incorporating a smearing width of 0.027 eV for the precise determination of spin dynamics. For optimal accuracy, we set the time step at $\Delta t = 0.1$ a.u. The laser pulses employed in our study were purposefully linearly polarized, specifically with an in-plane orientation, and operated at a carefully selected frequency. Throughout the entire process, we strictly adhered to adiabatic local spin density approximations (ALSDA) [8].

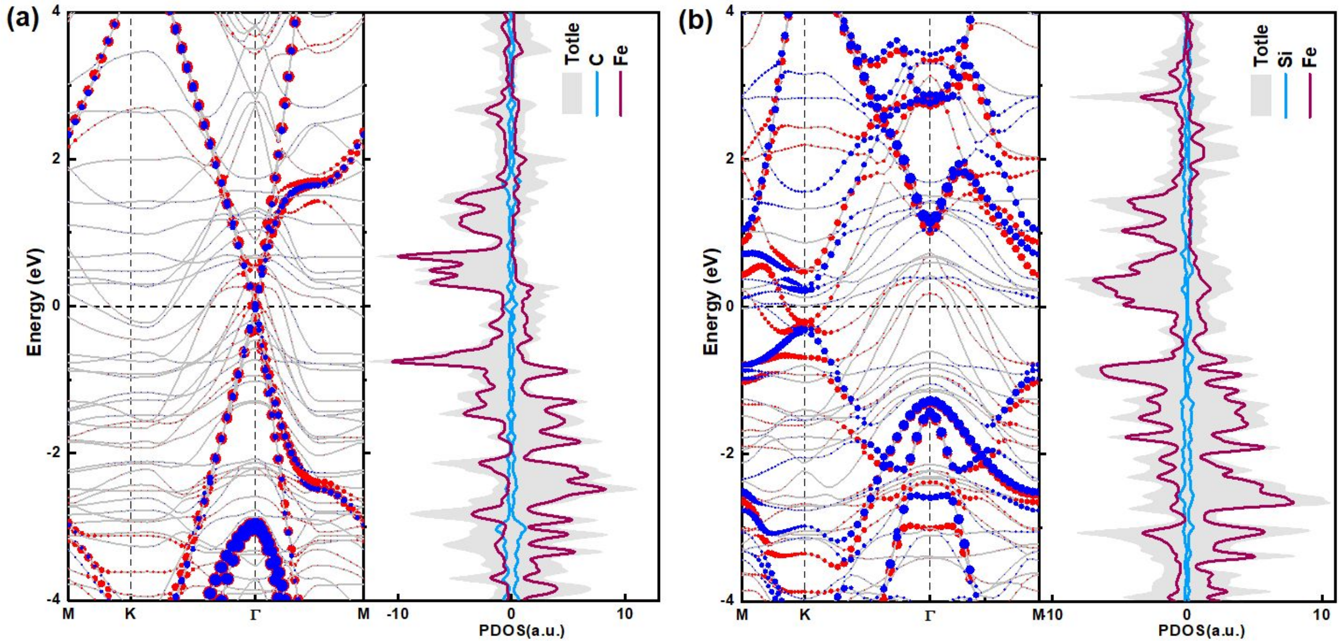

Figure S1. Projected band structure and PDOS: (a) graphene and (b) silicene dos of NM layer; the spin-up and spin-down are marked by red and blue spheres, respectively.

Table S1. Geometric structure parameters and initial magnetic moment. The mismatch of Lattice (ML) defined

ML= (L_{NM} -L_{FGT})/L_{FGT} . The positive and negative values of L represent the ratio between stretch/tensile and compressive forces of NM materials, respectively. M_{NM1}, M_{NM2}, M_{Fe1}, M_{Fe2}, M_{Fe3} stand for the local magnetic moment of NM and FGT, as shown in Figure 1. d is the distance between NM and FGT layers.

Structure	ML	$M_{NM1}(\mu_B)$	$M_{NM2}(\mu_B)$	$M_{Fe1}(\mu_B)$	$M_{Fe2}(\mu_B)$	$M_{Fe3}(\mu_B)$	$d(\text{Å})$
Graphene/FGT	5.55%	0.001	0.001	2.454	2.423	1.434	3.356
Silicene/FGT	-4.44%	-0.021	0.031	2.433	2.453	1.422	2.922

Table S2. Various frequencies of Laser pulse. The spin dynamics of Si/FGT and Gr/FGT under the influence of laser pulses given by FWHM=3.63 fs, power density = 4.8×10^{12} W/cm2 with different frequency, phonon energy and peak intensities.

Frequency (THZ)	Power density (W/cm2)	Phonon enerfy (eV)	FWHM (fs)	Fluence (mJ/cm2)
13.2	4.8E+12	0.1	3.6	16.2
26.3	4.8E+12	0.1	3.6	15.9
131.7	4.8E+12	0.5	3.6	12.1
790.1	4.8E+12	3.3	3.6	10.9
1053.5	4.8E+12	4.4	3.6	10.9
1316.8	4.8E+12	5.4	3.6	10.9

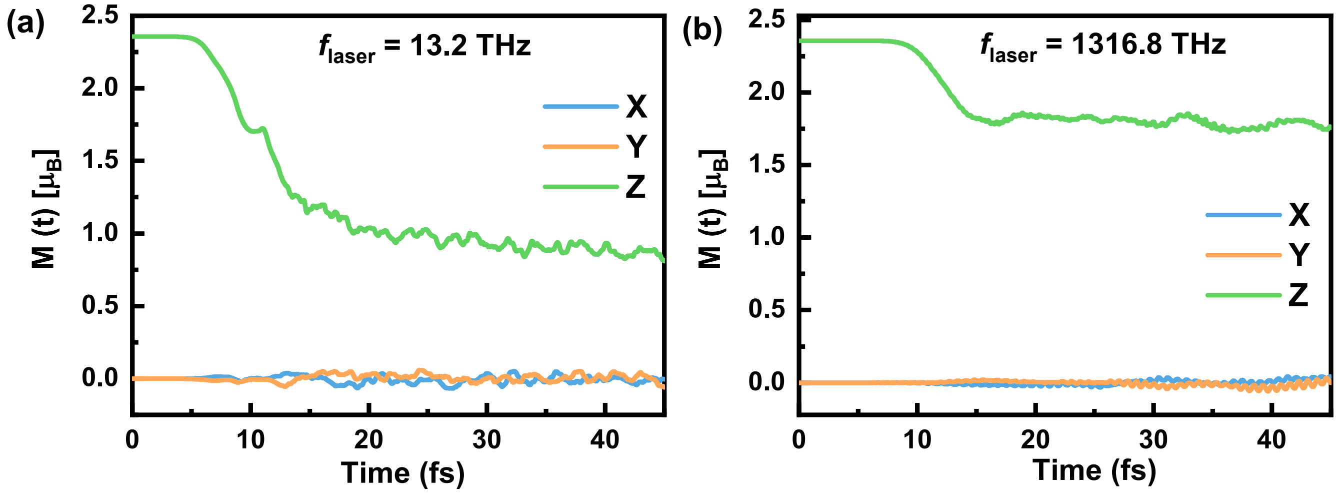

Figure S2. Ultrafast laser-induced ultrafast magnetization dynamics. Time evolution of the local magnetic moment in the x, y, and z directions for Fe1 at frequencies of 13.2 THz (a) and 1316.8 THz (b).

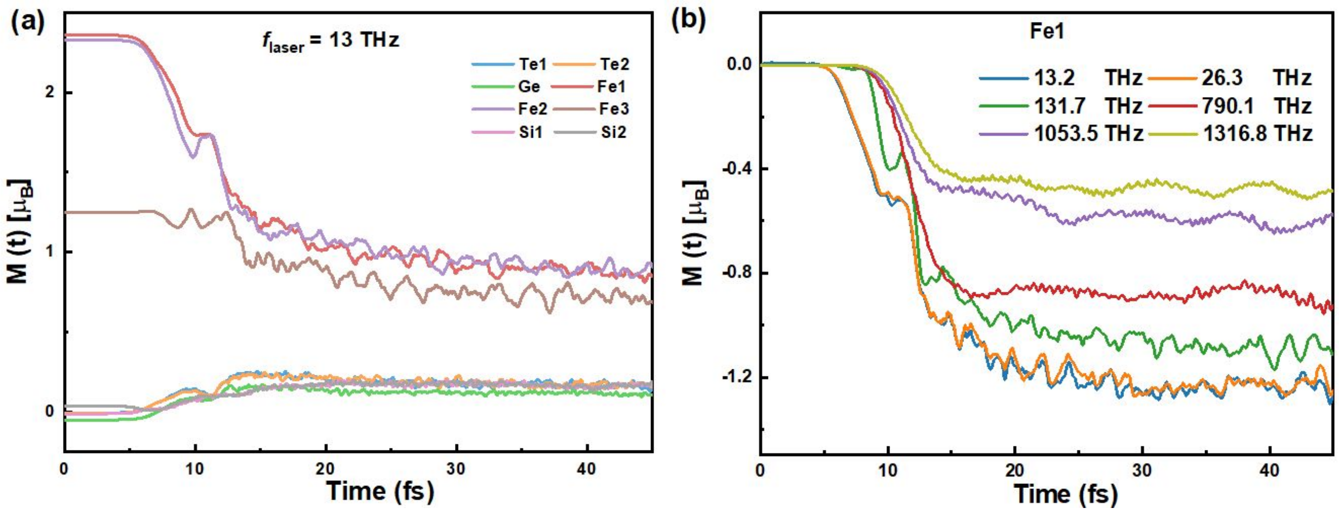

Figure S3. Ultrafast laser-induced magnetization dynamics. (a) Time evolution of the local magnetic moment of Fe, Ge, Te, and Si atoms. (b) Time dependent dynamics of the local magnetic for the Fe1 of the Gr/FGT in different pulse (13.2 THz, 26.3 THz, 131.7 THz, 790.1 THz, 1053.5 THz, and 1316.8 THz).

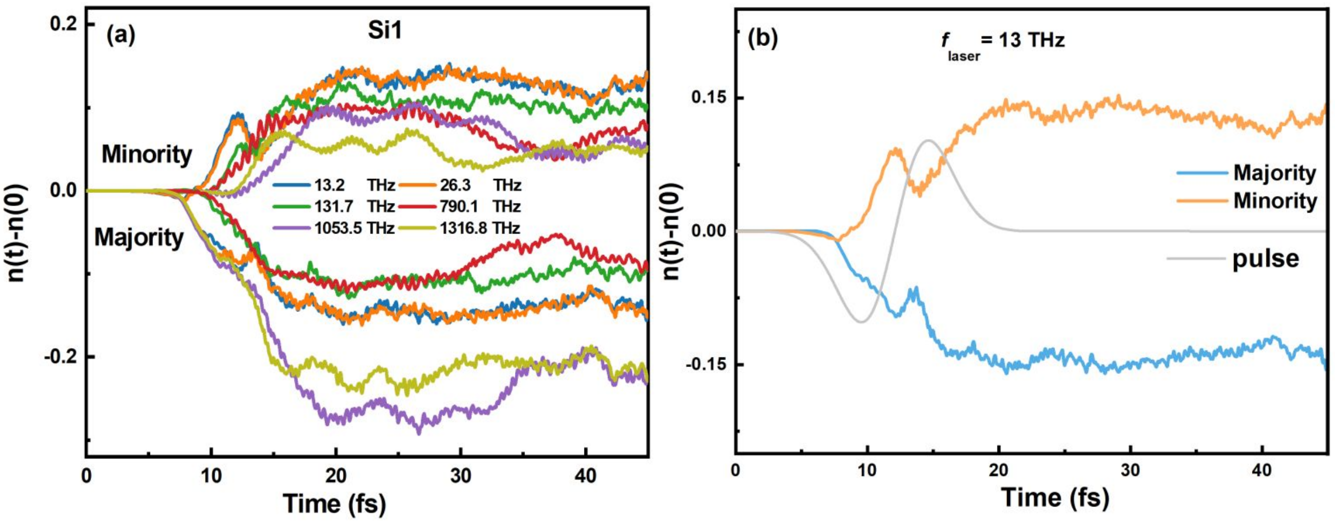

Figure S4. Time-dependent occupation dynamics. (a) The time dependent change of majority and minority occupations as a function of time (in fs) of Si1 atoms of the Si/FGT, which defined as n(t)-n(0); (b) The occupations of Si1 at the frequency 13.2 THz, and the laser pulse was plot by using the grey line.

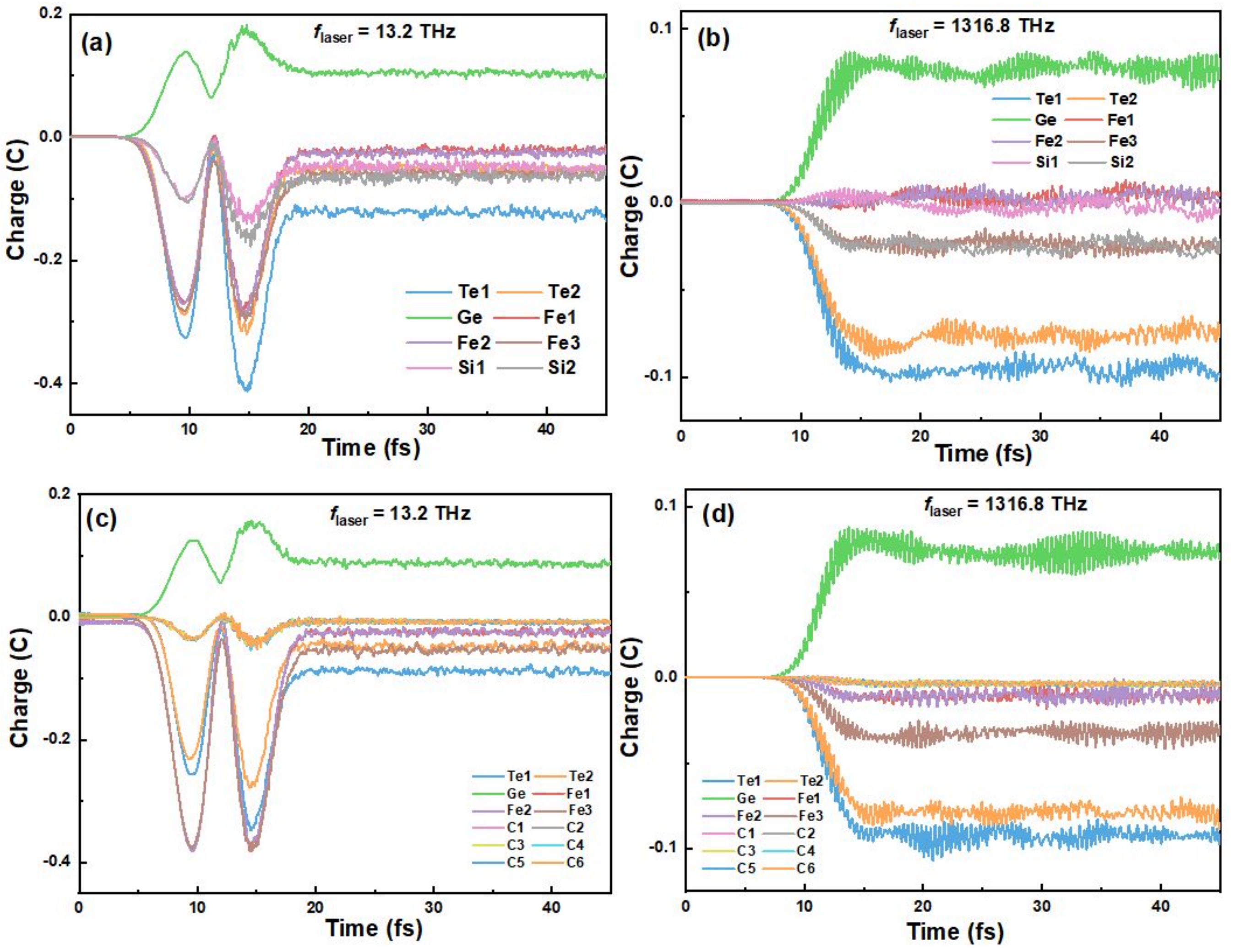

Figure S5. Time dependent charge dynamics. The charge density for Si/FGT in frequency of 13.2 THz (a) and 1316.8 THz (b), while for Gr/FGT in frequency of 13.2 THz (c) and 1316.8 THz (d).

Reference:

(1) Kresse, G.; Hafner, J. Ab initio molecular dynamics for liquid metals. *Phys. Rev. B* **1993**, *47* (1), 558.
(2) Perdew, J. P.; Burke, K.; Ernzerhof, M. Generalized gradient approximation made simple. *Phys. Rev. Lett.* **1996**, *77* (18), 3865-3868.
(3) Kresse, G.; Furthmüller, J. Efficient iterative schemes for ab initio total-energy calculations using a plane-wave basis set. *Phys. Rev. B* **1996**, *54* (16), 11169-11186.
(4) Blöchl, P. E. Projector augmented-wave method. *Phys. Rev. B* **1994**, *50* (24), 17953.
(5) Grimme, S.; Antony, J.; Ehrlich, S.; Krieg, H. A consistent and accurate ab initio parametrization of density functional dispersion correction (DFT-D) for the 94 elements H-Pu. *J. Chem. Phys.* **2010**, *132* (15), 154104.

(6) Grimme, S. Semiempirical GGA-type density functional constructed with a long-range dispersion correction. *J. Comput. Chem.* **2006**, *27* (15), 1787-1799.
(7) Dewhurst, J. K. S., S.; et al. Elk code. *elk.sourceforge.net* (accessed Oct 1, 2020).
(8) Von Barth, U.; Hedin, L. A local exchange-correlation potential for the spin polarized case. i. *J. Phys. C: Solid State Phys.* **1972**, *5* (13), 1629.
